# Supplementary material for: The pattern and burden of non-communicable diseases in armed conflict-exposed populations in Northeastern Nigeria
Source: PeerJ. 2025 Jan 17;13:e18520. doi: 10.7717/peerj.18520 (PMC11745130; doi:10.7717/peerj.18520)
Supplement: Supplemental Information 2 [file peerj-13-18520-s002.docx]

**Questionnaire**

**Socio-Demographics**

1)Age

(ii) 20–29 (iii) 30–39 (iv) 40–49 (v) 50–59 (vi) 60 and above

2)Tribe

3) (i) Fulani (ii) Hausa (iii) Bachama (iii) Kilba (iv) marghi

4)Sex/Gender

i) Male { } ii) Female { }

5)Marital status

(i) Married { } (ii) single { } (iii) Divorced { } (iv) widowed { } (v) separated { }

6.) family size………………

7)Educational status (strata of education)

i) No formal education { } (ii) primary school { } (iii) secondary { } (iv) tertiary { }

8)occupation

(i) Unemployed { } (ii) trader/ business { } (iii) civil servant ( iv) herder (v) farmer (vi) Hunter { } (vii) healthcare worker (viii) artisan { } (ix)others

9.) Average size of income per month (i) < N10,000 { } (ii) N10,000- N19,000 { }

(iii)N20,000 - N29,000 { } (iv) N30,000- N39,000 { } (v)N40,000 -N49000 { } (vi)

10). Do you have HealthCare insurance? (i) Yes { } (ii) No { }

11.) socioeconomic status (i) low income status (ii) middle-income status (iii) high income status

**Chronic diseases profile**

12) Which of this chronic disease condition do you have (self-reported)

1. Hypertension { } (ii) Diabetes (iii) arthritis (iv) stroke (v) (vi) cancer( type) (vii) asthma (viii) obesity (ix) COPD (x) ) chronic kidney disease (xi) chronic liver disease (xii) others

13.Do you attend follow up regularly? (i) Yes (ii) (ii) No

14.If no, why? (i) lack of transport fair or poor road network to the hospital (ii) ignorance that NCDs require routine follow up (iii) I don’t care attitude (iv) not necessary

15.Have you ever been admitted in the hospital due to any chronic illness before.?

16. How often do you come for follow up in every two months? (Frequency of hospital visits)

17) Are you being disabled, limited or have been prevented by your illness from undertaking your job, schooling or other activities of daily living like you used to do in the past? (i) Yes (ii) No

18) Health utility scale………………

19) How much were you spending averagely per month in the past (8 yrs ago) in buying your medication or treating your chronic illness ?………………….

20.) And how much are you now spending in buying the same medications? ………………..

21. Are you able to buy all your prescribed medicines regularly in the last 5yrs? (i) Yes (ii)No if no why?........................

22.Does any of your first-degree relative suffer from any of these chronic diseases?

i)Hypertension { } (ii) Diabetes (iii) arthritis (iv) stroke (v) (vi) cancer (vii) asthma (viii) obesity (ix) cancers

**Anthropometrics measurement**

23 Waist circumference ………… (ii) hip circumference (iii) waist hip ratio WHR……..

i) Blood pressure ………. b) BP…………. c) BP …………. Average BP………...

**NCDs (non-communicable disease) Risk profile**

24.Do you smoke**?** (i) Yes (ii) No if yes quantify number of sticks per day…………………. And for low long have you been smoking …………….

25.Do you use snuff? (i) Yes (ii) No

26.Do you drink Alcohol? (i) Yes (ii) No if yes quantify number of bottles per week…………………or quantify number of calabashes per week………………….

27.Do you take 5 serving of fruits on daily basis as recommended? (i) Yes (ii) No………

28.Have you lost a first degree relative due to complications of any of these diseases (verbal autopsy)? (i) diabetes (i) hypertension (iii) stroke (iv) heart attack

29.Approximate of Amount of salt consume you take per day/ adds raw salt to already prepared meals on the table (i) <5g/day……… (ii) 5g>/day………………...

**PHYSICAL ACTIVITY QUESTIONNAIRE**

30.Physical inactivity will be classified as the failure to meet WHO recommendations on physical activity for health, which are defined as engaging in at least 150 minutes of moderate-intensity activity per week or 75 minutes of vigorous-intensity activity per week

**Metabolic profile**

31). Blood sugar level- (i) Random blood sugar ……… (ii) Fasting blood sugar ……

32.) total Serum cholesterol…………….

**Social factors**

33) have you been displaced or affected indirectly or indirectly by the banditry, insurgency, kidnapping or farmer-herders clash affecting the country? a) Yes ………….. b) No

**34.Final diagnosis( made by doctors)**

**1. …………………..**  disease complications………………..

**2……………………… …………………………………**

**3………………………. ………………………………….**

**4…………………………. …………………………………**
