# Supplementary material for: The pattern and burden of non-communicable diseases in armed conflict-exposed populations in Northeastern Nigeria
Source: PeerJ. 2025 Jan 17;13:e18520. doi: 10.7717/peerj.18520 (PMC11745130; doi:10.7717/peerj.18520)
Supplement: Supplemental Information 3 [file peerj-13-18520-s003.docx]

**Multivariable Logistic and Multivariable Clog-Log Regression of NCD**

(1) (2) (3) (4)

**Abdominal obesity** **95% CI** **Hypertension** **95% CI** **Depression** **95% CI** **Diabetes**  **95% CI**

**aOR** **aOR IRR IRR**

----------------------------------------------------------------------------------------------------------------------------------------

main

Jada 1.00 [1.00,1.00] 1.00 [1.00,1.00] 1.00 [1.00,1.00] 1.00 [1.00,1.00]

Mubi 0.18*** [0.11,0.28] 0.82 [0.50,1.34] 4.78*** [2.51,9.12] 2.00 [0.82,4.86]

no 1.00 [1.00,1.00] 1.00 [1.00,1.00] 1.00 [1.00,1.00] 1.00 [1.00,1.00]

yes 1.94** [1.23,3.08] 1.03 [0.63,1.68] 1.76* [1.08,2.88] 0.33* [0.13,0.80]

nage=18 1.00 [1.00,1.00] 1.00 [1.00,1.00] 1.00 [1.00,1.00] 1.00 [1.00,1.00]

nage=38 2.08*** [1.46,2.96] 1.85** [1.21,2.83] 2.46*** [1.60,3.79] 5.71** [2.01,16.23]

nage=58 1.87* [1.12,3.14] 2.63*** [1.52,4.55] 1.70 [0.96,3.02] 11.92*** [3.27,43.42]

nage=78 1.04 [0.36,2.98] 4.39** [1.64,11.77] 0.38 [0.05,2.68] 4.56 [0.43,48.25]

female 1.00 [1.00,1.00] 1.00 [1.00,1.00] 1.00 [1.00,1.00] 1.00 [1.00,1.00]

male 0.41*** [0.29,0.59] 1.06 [0.71,1.58] 0.80 [0.56,1.14] 1.80 [0.88,3.66]

Divorced 1.00 [1.00,1.00] 1.00 [1.00,1.00] 1.00 [1.00,1.00] 1.00 [1.00,1.00]

Married 0.52 [0.21,1.30] 0.74 [0.27,2.02] 0.94 [0.26,3.37] 0.50 [0.09,2.74]

Separated 1.00 [1.00,1.00] 1.00 [1.00,1.00] 1.00 [1.00,1.00] 1.00 [1.00,1.00]

Single 0.60 [0.23,1.58] 0.40 [0.12,1.29] 1.02 [0.26,4.10] 0.14 [0.01,2.48]

Widowed 0.59 [0.20,1.76] 1.32 [0.42,4.19] 0.92 [0.23,3.67] 0.24 [0.03,1.71]

No formal educ~n 1.00 [1.00,1.00] 1.00 [1.00,1.00] 1.00 [1.00,1.00] 1.00 [1.00,1.00]

Primary school 1.07 [0.65,1.76] 0.92 [0.53,1.60] 0.93 [0.56,1.54] 0.90 [0.35,2.32]

Secondary 0.75 [0.50,1.14] 0.63 [0.39,1.00] 0.90 [0.56,1.45] 1.75 [0.75,4.06]

Tertiary 0.65 [0.41,1.03] 0.43** [0.23,0.78] 1.23 [0.75,2.04] 1.20 [0.46,3.17]

Artisan 1.00 [1.00,1.00] 1.00 [1.00,1.00] 1.00 [1.00,1.00] 1.00 [1.00,1.00]

Civil Servant 1.42 [0.63,3.20] 1.72 [0.66,4.48] 0.94 [0.41,2.14] 2.25 [0.79,6.47]

Farmer 0.93 [0.43,2.02] 1.39 [0.57,3.39] 1.60 [0.75,3.38] 1.06 [0.37,3.00]

Full time hous~e 0.87 [0.37,2.04] 1.79 [0.68,4.71] 1.40 [0.55,3.55] 3.22 [0.93,11.17]

Herder 0.72 [0.23,2.31] 1.45 [0.35,5.93] 1.00 [1.00,1.00] 1.61 [0.17,15.73]

Other 0.89 [0.38,2.07] 1.24 [0.47,3.27] 1.88 [0.80,4.41] 1.88 [0.61,5.81]

Student 0.51 [0.19,1.40] 0.50 [0.08,2.96] 0.71 [0.23,2.22] 1.00 [1.00,1.00]

Trader/Business 1.25 [0.59,2.63] 1.26 [0.52,3.07] 1.64 [0.78,3.42] 1.00 [1.00,1.00]

Low Income 1.00 [1.00,1.00] 1.00 [1.00,1.00] 1.00 [1.00,1.00] 1.00 [1.00,1.00]

Middle Income 0.82 [0.54,1.23] 0.88 [0.54,1.43] 0.95 [0.62,1.47] 0.54 [0.26,1.13]

Upper Income 2.01 [0.65,6.26] 4.36* [1.13,16.80] 2.41 [0.92,6.36] 2.73 [0.34,22.19]

no 1.00 [1.00,1.00] 1.00 [1.00,1.00] 1.00 [1.00,1.00] 1.00 [1.00,1.00]

yes 0.88 [0.45,1.70] 0.96 [0.47,1.97] 1.12 [0.58,2.17] 0.72 [0.24,2.14]

no 1.00 [1.00,1.00] 1.00 [1.00,1.00] 1.00 [1.00,1.00] 1.00 [1.00,1.00]

yes 0.51 [0.22,1.18] 0.51 [0.14,1.85] 1.67 [0.76,3.71] 0.82 [0.12,5.66]

no 1.00 [1.00,1.00] 1.00 [1.00,1.00] 1.00 [1.00,1.00] 1.00 [1.00,1.00]

yes 1.61 [0.65,3.98] 0.19 [0.02,1.66] 3.13* [1.31,7.49] 1.46 [0.31,6.79]

no 1.00 [1.00,1.00] 1.00 [1.00,1.00] 1.00 [1.00,1.00] 1.00 [1.00,1.00]

yes 1.12 [0.63,1.99] 1.66 [0.89,3.12] 0.50 [0.23,1.09] 0.26 [0.02,2.84]

no 1.00 [1.00,1.00] 1.00 [1.00,1.00] 1.00 [1.00,1.00] 1.00 [1.00,1.00]

yes 0.75 [0.24,2.31] 1.26 [0.38,4.13] 1.00 [1.00,1.00] 1.00 [1.00,1.00]

<5g/day 1.00 [1.00,1.00] 1.00 [1.00,1.00] 1.00 [1.00,1.00] 1.00 [1.00,1.00]

>5g/day 1.05 [0.79,1.40] 1.16 [0.83,1.63] 1.40 [0.99,1.97] 1.25 [0.68,2.30]

no 1.00 [1.00,1.00] 1.00 [1.00,1.00] 1.00 [1.00,1.00] 1.00 [1.00,1.00]

yes 1.19 [0.88,1.62] 0.90 [0.62,1.32] 1.16 [0.82,1.63] 1.65 [0.81,3.33]

no 1.00 [1.00,1.00] 1.00 [1.00,1.00] 1.00 [1.00,1.00] 1.00 [1.00,1.00]

yes 1.07 [0.70,1.63] 3.12*** [2.00,4.88] 1.30 [0.81,2.09] 2.96** [1.39,6.31]

no 1.00 [1.00,1.00] 1.00 [1.00,1.00] 1.00 [1.00,1.00] 1.00 [1.00,1.00]

yes 1.04 [0.60,1.79] 0.95 [0.52,1.73] 1.04 [0.59,1.84] 4.31*** [1.92,9.67]

----------------------------------------------------------------------------------------------------------------------------------------

n 972 972 929 807

chi2 143.74*** 110.77*** 158.06*** 102.47***

bic 1371.77 1120.57 911.96 476.93

Model vs empty F(30,972)= 143.74 F(30, 972) = 110.77; F(28, 929)= 158.06 F(27, 807)= 102.47

Model (Wald or LR) p < 0.001 p < 0.001 p < 0.001 p < 0.001

Goodness-of-fit Hosmer-Lemeshow: Hosmer-Lemeshow: Zero outcomes = 755 Zero outcomes = 758

Test F(8, 972) = 10.54; F(8,972)=16.02 Nonzero outcomes = 174 Nonzero outcomes = 49

p = 0.229 p = 0.042

Area under ROC 0.7393 0.7518

curve

----------------------------------------------------------------------------------------------------------------------------------------

Exponentiated coefficients; 95% confidence intervals in brackets

* p<0.05, ** p<0.01, *** p<0.001

**Appendix**

**Table A1: Logistic Regression of NCD**

(3) (4)

**Depression** **95% CI** **Diabetes**  **95% CI**

**aOR aOR**

----------------------------------------------------------------------------

main

Jada 1.00 [1.00,1.00] 1.00 [1.00,1.00]

Mubi 5.32*** [2.65,10.70] 2.05 [0.79,5.33]

no 1.00 [1.00,1.00] 1.00 [1.00,1.00]

yes 2.05* [1.17,3.58] 0.30* [0.12,0.80]

nage=18 1.00 [1.00,1.00] 1.00 [1.00,1.00]

nage=38 2.94*** [1.77,4.89] 6.37*** [2.13,19.04]

nage=58 1.85 [0.95,3.58] 13.74*** [3.57,52.93]

nage=78 0.33 [0.05,2.41] 4.77 [0.44,51.46]

female 1.00 [1.00,1.00] 1.00 [1.00,1.00]

male 0.76 [0.49,1.16] 1.89 [0.88,4.04]

Divorced 1.00 [1.00,1.00] 1.00 [1.00,1.00]

Married 1.06 [0.24,4.62] 0.44 [0.05,3.81]

Separated 1.00 [1.00,1.00] 1.00 [1.00,1.00]

Single 1.22 [0.24,6.12] 0.12 [0.00,3.07]

Widowed 1.01 [0.20,5.06] 0.20 [0.02,2.10]

No formal educ~n 1.00 [1.00,1.00] 1.00 [1.00,1.00]

Primary school 0.92 [0.49,1.73] 0.85 [0.32,2.29]

Secondary 0.89 [0.50,1.58] 1.91 [0.79,4.65]

Tertiary 1.31 [0.70,2.45] 1.29 [0.44,3.82]

Artisan 1.00 [1.00,1.00] 1.00 [1.00,1.00]

Civil Servant 0.92 [0.35,2.43] 2.33 [0.74,7.32]

Farmer 1.77 [0.71,4.39] 1.17 [0.39,3.53]

Full time hous~e 1.57 [0.52,4.81] 3.75* [1.03,13.67]

Herder 1.00 [1.00,1.00] 1.83 [0.15,22.33]

Other 2.14 [0.77,5.98] 2.14 [0.60,7.67]

Student 0.64 [0.18,2.25] 1.00 [1.00,1.00]

Trader/Business 1.88 [0.77,4.57] 1.00 [1.00,1.00]

Low Income 1.00 [1.00,1.00] 1.00 [1.00,1.00]

Middle Income 0.93 [0.55,1.56] 0.55 [0.25,1.23]

Upper Income 2.96 [0.77,11.46] 2.96 [0.41,21.51]

no 1.00 [1.00,1.00] 1.00 [1.00,1.00]

yes 1.22 [0.54,2.76] 0.69 [0.20,2.36]

no 1.00 [1.00,1.00] 1.00 [1.00,1.00]

yes 1.55 [0.60,3.99] 0.82 [0.10,6.86]

no 1.00 [1.00,1.00] 1.00 [1.00,1.00]

yes 4.02* [1.25,12.89] 1.20 [0.24,5.94]

no 1.00 [1.00,1.00] 1.00 [1.00,1.00]

yes 0.42 [0.17,1.01] 0.28 [0.03,2.58]

no 1.00 [1.00,1.00] 1.00 [1.00,1.00]

yes 1.00 [1.00,1.00] 1.00 [1.00,1.00]

<5g/day 1.00 [1.00,1.00] 1.00 [1.00,1.00]

>5g/day 1.48 [0.99,2.21] 1.28 [0.66,2.48]

no 1.00 [1.00,1.00] 1.00 [1.00,1.00]

yes 1.22 [0.81,1.85] 1.70 [0.79,3.68]

no 1.00 [1.00,1.00] 1.00 [1.00,1.00]

yes 1.40 [0.79,2.47] 3.11** [1.42,6.80]

no 1.00 [1.00,1.00] 1.00 [1.00,1.00]

yes 1.09 [0.55,2.20] 5.12*** [2.11,12.42]

----------------------------------------------------------------------------

n

chi2 144.46*** 87.27***

bic 911.69 477.45

Model vs empty F(28, 929)= 144.46 F(27, 807)= 87.27

Model (Wald or LR) p < 0.001 p < 0.001

----------------------------------------------------------------------------

Exponentiated coefficients; 95% confidence intervals in brackets

* p<0.05, ** p<0.01, *** p<0.001
